# Cognitive impairment in vestibular migraine: a PubMed-based systematic review and meta-analysis

**DOI:** 10.3389/fneur.2026.1818747

**Published:** 2026-09-03

**Authors:** Meiqi Di, Lingling Hu, Chaosheng Li, Xiaojing Yin, Shuhua Gui, Likun Han

**Affiliations:** Department of Neurology, Affiliated Hospital of Jiangnan University, Wuxi, Jiangsu, China

**Keywords:** cognitive impairment, executive function, global cognition, stroop performance, vestibular migraine

## Abstract

**Background:**

Vestibular migraine (VM) is a leading cause of recurrent vertigo, and many patients describe cognitive symptoms such as “brain fog,” slowed thinking, and forgetfulness; however, the magnitude and consistency of objectively measured cognitive impairment in VM have not been clearly quantified. We therefore conducted a PubMed-only systematic review (2010–2025; English; humans) of observational studies reporting quantitative behavioral cognitive outcomes or explicit impairment thresholds in adults with VM. Two reviewers independently extracted data and assessed the risk of bias using ROBINS-I. Continuous outcomes were pooled as standardized mean differences (SMD; Hedges g; random-effects REML), while the prevalence of study-defined cognitive impairment in VM cohorts was synthesized using the Freeman-Tukey transformation; when required, medians and measures of dispersion were converted to means and standard deviations.

**Results:**

Six studies met the inclusion criteria, of which four contributed to the primary meta-analysis (VM *n* = 208; healthy controls [HC] *n* = 180). Compared with HC, adults with VM demonstrated poorer global cognition (SMD −0.92, 95% CI −1.13 to −0.70; I^2^ = 8%), with a prediction interval of −1.38 to −0.45. Executive function, indexed by Stroop performance in two studies, also suggested a large deficit (SMD + 1.55, 95% CI 0.77–2.33; I^2^ = 79%), though this pooled estimate should be interpreted cautiously as an exploratory signal due to the high heterogeneity driven by differing Stroop metrics. Across VM cohorts, the pooled prevalence of study-defined cognitive impairment was 0.74 (95% CI 0.66–0.81; I^2^ = 0%).

**Conclusion:**

Based on this focused, single-database rapid review, current evidence suggests adults with VM may exhibit moderate-to-large decrements in global cognition and executive control, supporting the consideration of cognitive screening and multidisciplinary management in routine VM care. Future work should prioritize standardized cognitive outcomes and mechanistic studies to clarify causal pathways and improve clinical translation.

**Systematic review registration:**

https://inplasy.com/projects/, Identifier, INPLASY2025100115.

## Introduction

Vestibular migraine (VM) is among the most common causes of recurrent vertigo in adults and represents a substantial source of disability (1). Beyond dizzy spells and migrainous headache, many patients describe cognitive complaints such as ‘brain fog’, difficulty concentrating, slowed processing, and memory lapses. Importantly, VM represents a substantial burden beyond vestibular symptoms alone, with considerable negative effects on health-related quality of life and daily functioning. Previous studies have demonstrated that patients with VM frequently experience psychiatric comorbidities, particularly anxiety and depressive symptoms, which may further amplify symptom burden and negatively influence cognitive performance (2, 3). These complaints are clinically important because they can affect driving, work productivity, fall risk, and adherence to vestibular rehabilitation; however, despite a growing clinical impression, the extent to which VM is associated with measurable cognitive impairment, on standardized tests rather than symptoms alone, remains uncertain.

Individual studies have reported reduced scores on global screening measures (eg, Montreal Cognitive Assessment [MoCA] or Addenbrooke’s Cognitive Examination-Revised [ACE-R]) and poorer performance on executive tasks such as the Stroop Color-Word test (4–6). Other reports, however, have found minimal differences or have emphasized that migraine itself, irrespective of vestibular symptoms, may carry small cognitive effects, which complicates attribution specifically to VM. In parallel, resting-state functional MRI studies in VM highlight alterations in attention, frontoparietal, and visual networks that plausibly link vestibular processing to cognition, but these imaging papers typically lack behavioral cognitive endpoints and therefore cannot address clinical effect sizes (7, 8).

Beyond network-level functional alterations, accumulating evidence in migraine biology implicates neuroimmune activation and inflammatory pathways. Peripheral cytokine/adipokine signatures and broader immune dysregulation have been reported in migraine and may contribute to attack recurrence and chronification, with potential downstream effects on hippocampal and frontoparietal circuits that subserve memory and executive control (9–11). Vestibular migraine likely shares core trigeminovascular mechanisms while additionally engaging vestibulo-cerebellar and vestibulo-thalamo-cortical pathways; within this framework, inflammation-mediated sensitization across these systems represents a plausible biological substrate for the cognitive symptoms frequently reported by patients with VM (12, 13).

To address this gap, we conducted a PubMed-based rapid systematic review and meta-analysis of behavioral cognitive outcomes in adults with VM. Our primary objective was to evaluate between-group differences in cognitive performance; secondary objectives were to evaluate executive dysfunction (Stroop performance) and summarize the proportion of patients meeting study-defined cognitive impairment thresholds. Single-arm VM cohorts were included only for the secondary prevalence synthesis and were not used for between-group effect-size pooling.

## Methods

### Study guideline

The present meta-analysis was conducted in accordance with the Preferred Reporting Items for Systematic Reviews and Meta-Analyses (PRISMA) guidelines. The protocol was registered in INPLASY (registration number: INPLASY2025100115) and is available at https://inplasy.com/?s=2025100115.

This review followed PRISMA 2020 reporting guidance (14), and the completed PRISMA 2020 checklist is provided in Supplementary material S1. A focused, single-database rapid review protocol (PubMed only) was prespecified to deliver a behavior-focused synthesis of cognition in VM; this methodological constraint was heavily weighted when interpreting the certainty and completeness of the evidence base. No ethics approval was required.

### Eligibility criteria

Population: Adults (≥18 years) diagnosed with VM or probable VM according to the *International Classification of Headache Disorders* (ICHD-3/ICHD-3β) and/or Bárány Society criteria were eligible. According to these criteria, VM diagnosis requires at least five episodes of vestibular symptoms of moderate or severe intensity lasting 5 min to 72 h, a current or previous history of migraine with or without aura, and migraine features (migraine-type headache, photophobia and phonophobia, or visual aura) during at least 50% of vestibular episodes. Probable VM requires recurrent vestibular symptoms with only one of the migraine-related features or a history of migraine without fulfilling all definite VM criteria.

Designs: Observational comparative studies (case–control, cross-sectional, cohort) and population surveys with analyzable data.

Comparators: Healthy controls (HC) or non-VM clinical controls for between-group analyses; a comparator was not required for single-arm estimates of the prevalence of cognitive impairment.

Outcomes (behavioral cognition only):

- Continuous: Standardized cognitive test scores (eg, ACE-R, MoCA, MMSE, Stroop, Trail-Making).
- Dichotomous: numbers meeting study-defined impairment thresholds (eg, ACE-R cutoffs). Imaging-only papers (e.g., rs-fMRI connectivity studies) without behavioral cognitive outcomes were excluded from quantitative pooling. Case reports/series (<10 VM participants), editorials, and non-English studies were also excluded.

### Information sources and search strategy

PubMed was searched from January 1, 2010 to September 6, 2025 (Humans; English) using the following string in Title/Abstract fields: (“vestibular migraine” OR “migrainous vertigo”) AND (cognition OR cognitive OR memory OR attention OR “executive function” OR MoCA OR MMSE OR “Addenbrooke*” OR ACE-R OR “Trail Making” OR Stroop OR antisaccade). Reference lists of eligible articles were also hand-searched to identify additional relevant studies.

### Study selection

Two reviewers independently screened titles and abstracts, followed by full-text assessment against the eligibility criteria. Disagreements were resolved through discussion. Imaging-only studies were documented for qualitative context but were excluded from the quantitative synthesis. Single-arm VM cohorts were included only for the secondary prevalence synthesis of study-defined cognitive impairment and were not used for between-group effect size pooling.

### Data extraction and data items

Using a standardized extraction form, two reviewers independently collected information on study design, setting/country, sample sizes, diagnostic criteria, participant demographics, testing state (ictal vs. interictal), comparator type, cognitive outcome definitions, and numerical outcome data (means/SDs for continuous outcomes; events/total for dichotomous outcomes). To avoid double-counting, we selected one effect per study for continuous outcomes using a prespecified hierarchy: ACE-R or MoCA (global cognition) was prioritized; if unavailable, MMSE was used, and for executive function, we preferentially extracted Stroop metrics. When studies reported medians with measures of dispersion, values were converted to means and SDs using the Wan/Luo methods (IQR to mean/SD) for median and interquartile range data and Hozo formulas (min-max to mean/SD) for median and range data, as appropriate (15, 16). For outcomes with inverse scaling (eg, longer completion time indicating poorer performance), directions were harmonized so that negative SMD values favored healthy controls (HC) for global cognition and positive SMD values favored HC for Stroop (ie, higher time indicating worse performance in VM).

### Risk of bias in individual studies

Non-randomized studies were appraised using ROBINS-I across seven domains (confounding; selection of participants; classification of interventions/exposures; deviations from intended interventions; missing data; outcome measurement; and selective reporting) (17). Judgments (Low/Moderate/Serious/Critical/No information) were made independently by two reviewers and finalized by consensus. Domain-level summaries were visualized using the “*robvis*” tool.

### Effect measures

For continuous outcomes, standardized mean differences (SMD; Hedges g) were pooled using random-effects models with restricted maximum likelihood (REML) estimation of *τ*^2^ (18). We report pooled effects with 95% confidence intervals (CI), together with *I*^2^ and *τ*^2^ to quantify heterogeneity, and we present a prediction interval for the primary outcome. For single-arm dichotomous prevalence estimates, we used “*metaprop*” with Freeman-Tukey double-arcsine transformation and a random-effects model (REML) (19). Unit-of-analysis was the study level; if multiple eligible instruments were reported in one study, the prespecified hierarchy was applied to select a single global cognition measure, while Stroop outcomes were analyzed separately.

### Synthesis methods

REML was used. Heterogeneity was summarized using *I*^2^ and *τ*^2^, and a prediction interval is shown for the primary outcome. Small-study effects were not formally tested, given the limited number of studies per analysis. All analyses were conducted in R (version ≥4.2) using the “*meta*” and “*metafor*” packages.

### Sensitivity analyses

Sensitivity analyses included: (1) excluding studies requiring median-to-mean conversion; (2) leave-one-out analyses; and (3) excluding studies rated as Serious risk in any ROBINS-I domain.

### Certainty assessment

A formal GRADE assessment was not performed because the available evidence was limited and heterogeneous, and the overall certainty should therefore be interpreted as low-to-moderate.

## Results

### Study selection and characteristics

**Figure 1:**
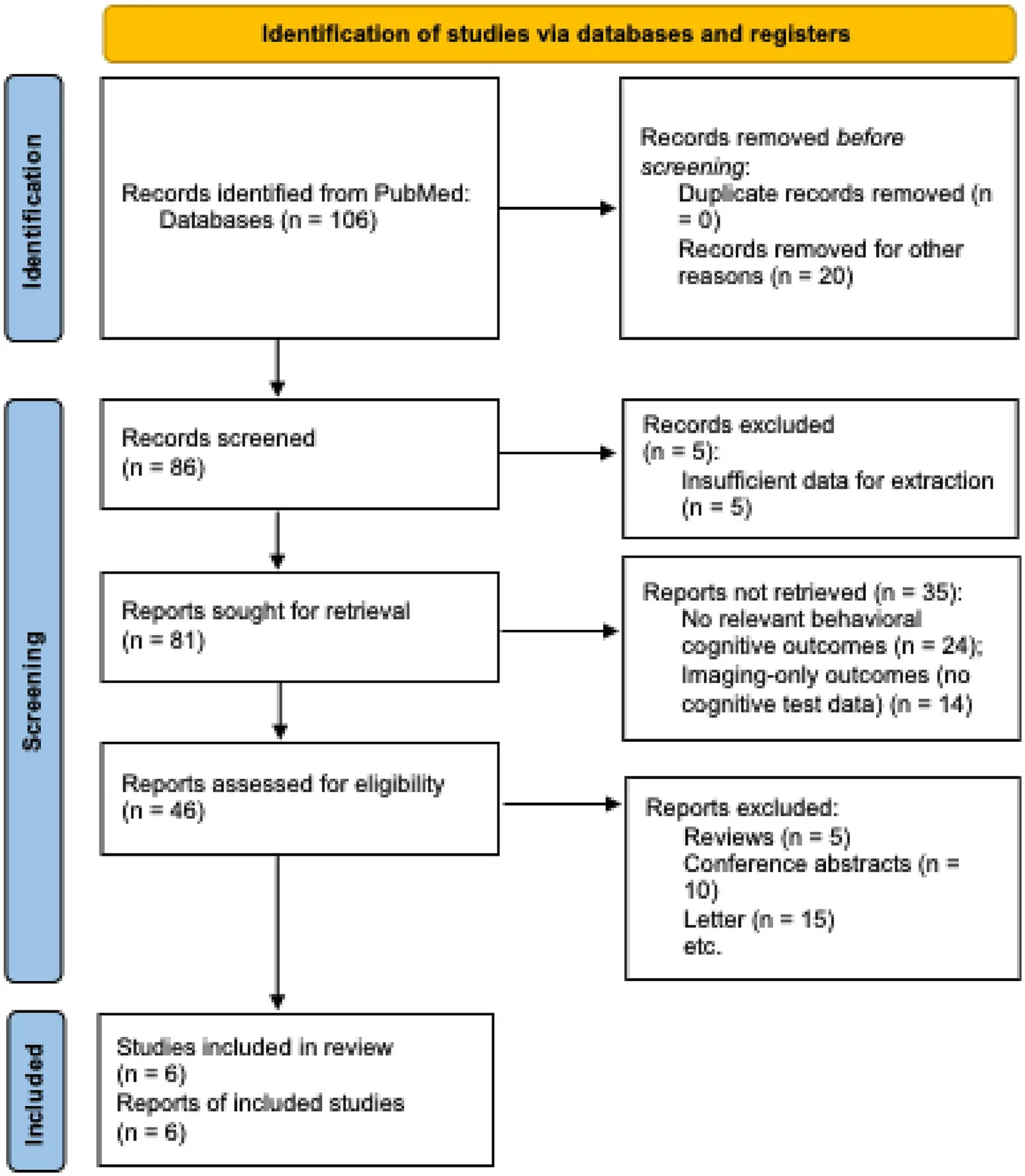
PRISMA flow diagram.

The study selection process is shown in the PRISMA flow diagram (Figure 1). The search identified 6 eligible studies for qualitative synthesis, of which 4 contributed data to the primary meta-analysis of global cognition (VM *n* = 208; HC *n* = 180), 2 contributed to the executive-function analysis based on Stroop performance (VM *n* = 82; HC *n* = 81), and 2 contributed to the prevalence analysis of cognitive impairment (VM total *n* = 139). Study designs consisted predominantly of clinic-based case–control or cross-sectional comparisons conducted in Turkey and China, together with one population-based survey from the United States. Most clinic-based studies assessed participants during the interictal period and applied standard diagnostic criteria, including those of the International Classification of Headache Disorders and/or the Bárány Society. Cognitive assessment instruments varied across studies but included established global and domain-specific measures, such as ACE-R, MoCA, MMSE, and Stroop variants. Consolidated baseline characteristics of the included studies are presented in Table 1.

**Table 1:** Baseline characteristics.

Study (Year)	Country	Design	Groups (VM/HC)	Age (VM vs. HC)	Female (%)	Cognitive measure(s)	Testing state	Diagnostic criteria
Balci et al. (20)	Turkey	Case–control, 3-arm^$^	32/31	41.3 ± 9.5 vs. 37.6 ± 7.2	matched	Stroop; BEST	Interictal	Clinical VM
Demirhan et al. (21)	Turkey	Case–control, 3-arm^$^	19/21	46.9 ± 6.8 vs. 51.1 ± 5.2	79/67	MMSE, Reading Span, Stroop, JLO, TMT	Interictal	Bárány/ICVD
Zhang et al. (22)	China	Cross-sectional, 3-arm^$^	78/79	51.8 ± 11.6 vs. 49.0 ± 15.0	86/72	ACE-R (plus BAEP)	Mixed (21 interictal; 57 ictal)	ICHD-III
Lu et al. (23)	China	Case–control	50/50	49.7 ± 9.5 vs. 50.4 ± 10.0	60/56	MoCA, MMSE, RBANS, TMT, Stroop; antisaccade	Interictal	ICHD-3 beta
Sun et al. (24)	China	Case–control	61/30	47 (median) vs. 45	93/87	ACE-R (incl. MMSE)	Interictal	ICHD + Bárány
Preysner et al. (25)*	USA	Population survey	VM 1,648 (5.1% of 32,047)	mean 43.4 y	69	Self-reported cognitive difficulty	Survey	Symptom-based case definition

*Survey contributed to qualitative context and risk-of-bias summary but not to continuous SMD pooling. $For multi-arm studies, only the VM and healthy control groups included in the present synthesis are shown. Sex distribution is reported as women/men unless otherwise specified. VM, vestibular migraine; HC, healthy control; ACE-R, Addenbrooke’s Cognitive Examination-Revised; MoCA, Montreal Cognitive Assessment; MMSE, Mini-Mental State Examination; RBANS, Repeatable Battery for the Assessment of Neuropsychological Status; TMT, Trail-Making Test; JLO, Judgment of Line Orientation; ICVD, International Classification of Vestibular Disorders; ICHD, International Classification of Headache Disorders.

### Risk of bias within studies

**Figure 2:**
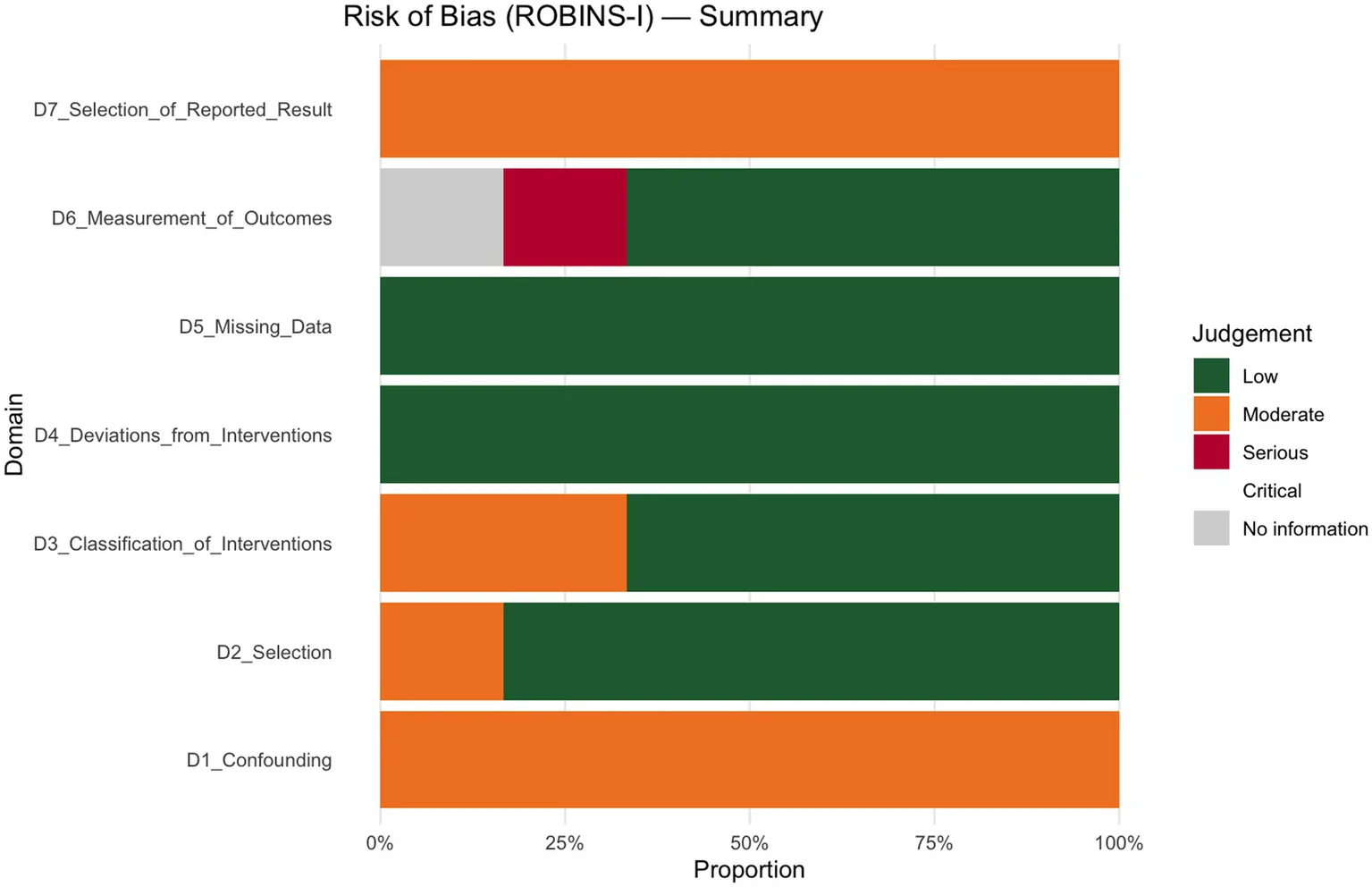
ROBINS-I domain-level risk-of-bias summary plot of the included studies.

ROBINS-I judgments were predominantly Low to Moderate across domains, with Low risk commonly assigned for missing data, deviations from intended exposures, and outcome measurement when objective cognitive tests were used. One population survey that relied on self-reported cognition was judged Serious for outcome measurement, and one study was rated as No information for that domain. The domain-level summary is shown in Figure 2.

### Global cognitive performance (VM vs. HC)

**Figure 3:**
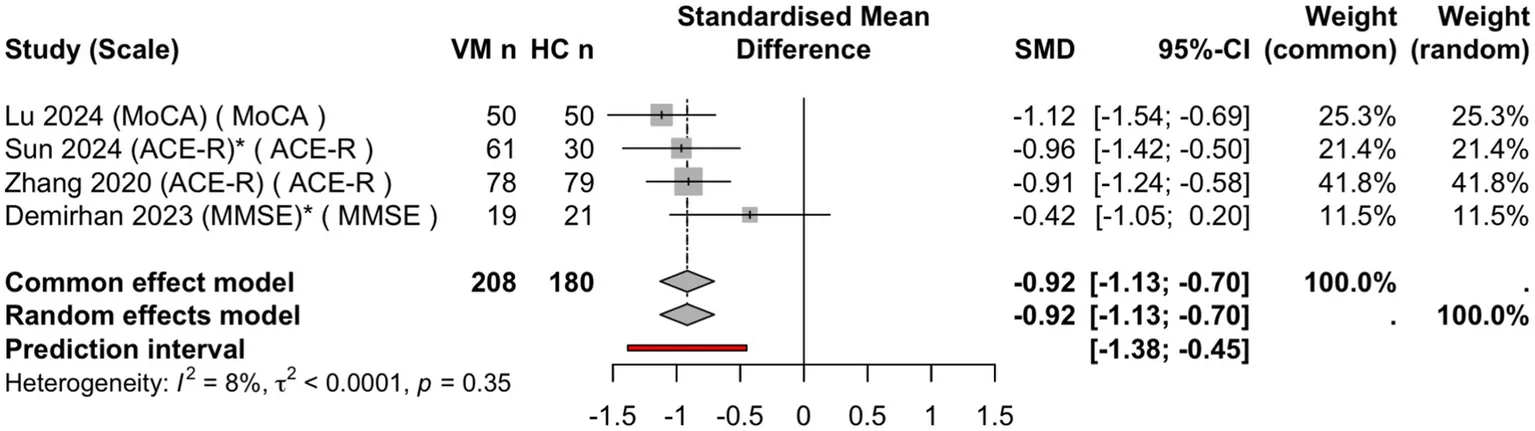
Random-effects meta-analysis of global cognitive performance expressed as standardized mean differences (SMD; Hedges’ *g*). Negative values indicate poorer cognitive performance in vestibular migraine (VM) compared with healthy controls (HC).

Across four studies using ACE-R, MoCA, or MMSE, global cognition was consistently worse in VM than in healthy controls. The random-effects model yielded a pooled SMD of −0.92 (95% CI −1.13 to −0.70), with low heterogeneity (*I*^2^ = 8%; τ^2^ < 0.0001; *p* = 0.35). The prediction interval (−1.38 to −0.45) suggests that future comparable studies would be expected to observe a negative effect of similar magnitude (Figure 3).

### Executive function (Stroop)

**Figure 4:**
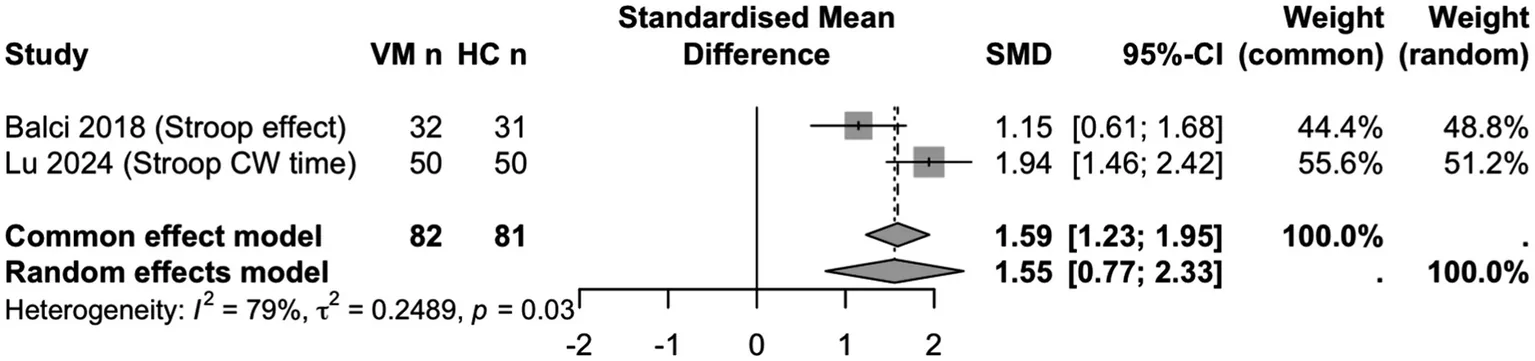
Random-effects meta-analysis of executive function assessed by Stroop performance, expressed as standardized mean differences (SMD; Hedges’ *g*). Positive values indicate slower or worse performance in participants with vestibular migraine (VM) compared with healthy controls (HC).

Two studies using Stroop interference/time indices demonstrated a large impairment in VM, with a pooled SMD of +1.55 (95% CI 0.77–2.33) and substantial heterogeneity (*I*^2^ = 79%; τ^2^ = 0.249; *p* = 0.03; Figure 4).

### Proportion with cognitive impairment in VM

**Figure 5:**
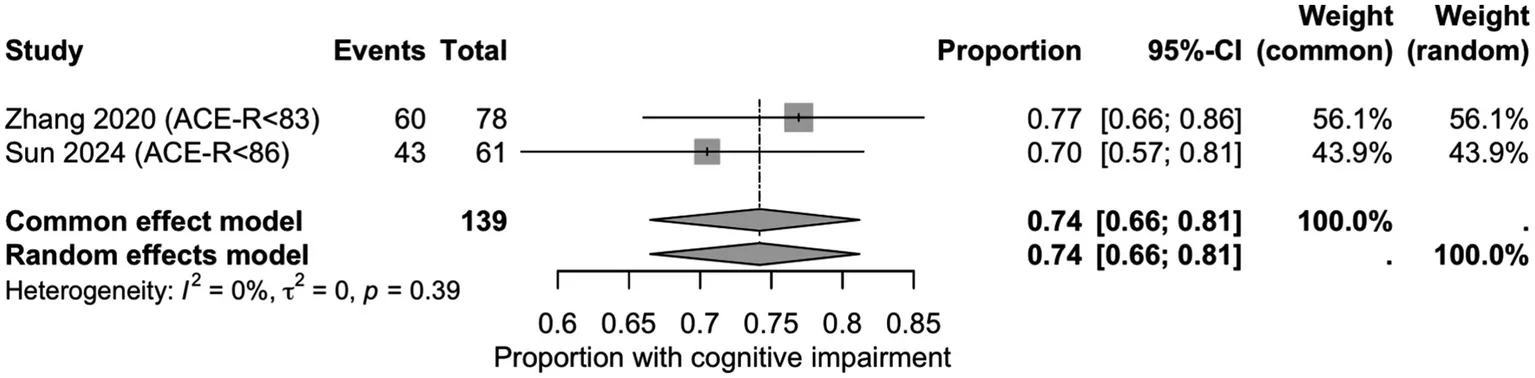
Random-effects meta-analysis of the proportion of patients with vestibular migraine (VM) meeting study-defined cognitive impairment thresholds. CI indicates confidence interval.

Two ACE-R-based cohorts reported cognitive impairment using study-specific cutoffs (<83 and <86). The pooled proportion meeting impairment criteria was 0.74 (95% CI 0.66–0.81), with no heterogeneity (*I*^2^ = 0%; τ^2^ = 0; *p* = 0.39; Figure 5).

### Sensitivity analyses

Full sensitivity results are provided in Supplementary material S2. Overall, the primary finding for global cognition was robust: excluding studies requiring median-to-mean/SD conversion yielded a similar, slightly larger deficit (SMD ≈ −0.99; I^2^ ≈ 0%). Leave-one-out analyses produced pooled effects ranging from approximately −0.84 to −0.98, with direction and statistical significance unchanged. Excluding studies with any ROBINS-I domain rated “Serious” did not affect the primary result because none of these studies contributed to the continuous meta-analysis. For executive function (Stroop), the pooled effect remained large despite heterogeneity, with single-study leave-one-out estimates (20) only vs. [Lu et al. (23) only] ranging from SMD ≈ +1.15 to +1.94. Small-study effects were not evident on Egger’s test for the primary outcome (k = 4), although statistical power was limited.

## Discussion

This focused synthesis indicates that adults with VM perform worse than healthy peers on standardized measures of global cognition, with a pooled deficit approaching one standard deviation (SMD −0.92) (20). Executive control, indexed by Stroop performance, shows an even larger impairment (SMD + 1.55), which is consistent with slowed inhibitory control and greater susceptibility to interference (26). However, this pooled effect size must be interpreted cautiously. Because the underlying studies utilized non-identical Stroop implementations (interference indices versus total completion time), the resulting quantitative estimate carries substantial heterogeneity and serves primarily as a preliminary, exploratory indicator of slowed inhibitory control and greater susceptibility to interference, rather than a definitive measurement. Although the included studies used different instruments (ACE-R, MoCA, MMSE; various Stroop variants), the direction of effect was uniform, and heterogeneity in the primary analysis was low. In this context, the results quantify a clinical impression frequently reported by patients—“brain fog,” slowed thinking, and forgetfulness—and translate these complaints into effect sizes that are likely to be both statistically and clinically meaningful (6).

The pattern of deficits aligns with proposed vestibulo-cognitive mechanisms. VM engages networks that are central to attention, multisensory integration, and top-down control (dorsal attention, frontoparietal, and visual networks) (27). Disturbance within these circuits could reduce the efficiency of sensory reweighting and attentional set-shifting, manifesting as poorer performance on global screening measures and on tasks requiring rapid inhibition (Stroop) (28). Clinically, this profile helps explain the difficulties that many VM patients report with driving, crowded environments, and high-load dual-tasking—situations that demand rapid integration of vestibular, visual, and proprioceptive cues under effective executive oversight.

In VM specifically, narrative reviews and neuroimaging studies suggest altered connectivity and/or functional activity within vestibular, pain and attentional networks, including cerebellar, insular, thalamic and frontoparietal nodes (13, 29–31). In this context, inflammatory and neuropeptide pathways, particularly CGRP-related signaling, may influence these circuits, which could lower the threshold for vestibular symptom expression and compromise executive control under conditions of high sensory load. Although the current mechanistic evidence remains indirect, future VM cohorts that incorporate inflammatory biomarkers alongside standardized cognitive endpoints may help determine whether inflammatory activity plays a mediating role in cognitive impairment or instead primarily reflects overall disease severity.

Neuroinflammation provides an additional biologically plausible link between VM and cognitive dysfunction. Contemporary migraine models extend beyond purely vascular explanations and emphasize trigeminal nociceptive signaling accompanied by immune and glial activation, with inflammatory mediators associated with symptom burden and, in some reports, with chronification (10, 11, 32, 33). In principle, sustained or recurrent inflammatory signaling could alter synaptic efficiency and neurovascular coupling within regions critical for attention and memory (eg, the prefrontal cortex and hippocampus), thereby contributing to subjective “brain fog” and to measurable deficits on global cognitive screening measures and inhibition-based tasks.

The prevalence synthesis suggests that approximately three-quarters of clinic-based VM samples meet study-defined thresholds for cognitive impairment (34). This estimate warrants cautious interpretation, because it represents an unadjusted, crude prevalence estimate. Zhang et al. excluded patients with clinically significant anxiety or depression, whereas Sun et al. included patients with psychiatric comorbidities and demonstrated that HADS-D were significantly associated with poorer ACE-R performance. Furthermore, neither study statistically adjusted the overall prevalence estimates for other important potential confounders, including sleep disturbance and prophylactic migraine medication use. Therefore, the observed prevalence likely reflects the combined effects of VM disease burden, psychiatric comorbidity, medication exposure, and other clinical factors rather than an isolated effect of VM pathology alone. Indeed, primary data from Sun et al. demonstrates a clear correlation between depression severity and cognitive decline in VM. Consequently, this 74% estimate likely reflects the cumulative clinical burden of VM and its common psychiatric comorbidities, rather than the isolated effect of the migraine biology alone. Furthermore, the variability in diagnostic cutoffs (ACE-R < 83 vs. <86) carries significant clinical implications and likely contributes to an overestimation of this figure. Utilizing a higher threshold (<86) increases screening sensitivity but compromises specificity. In a highly symptomatic, clinic-based population, applying this looser threshold risks misclassifying state-dependent, transient “brain fog” or fatigue as a stable cognitive impairment, thereby artificially inflating the overall prevalence estimate. Even so, the available evidence indicates that cognitive involvement is common in VM and therefore merits routine clinical attention. In practice, incorporating a brief cognitive screen (eg, MoCA or ACE-R) into VM visits could help identify patients who may benefit from closer monitoring, driving and occupational counseling, and individualized rehabilitation strategies (35).

Methodologically, broader or symptom-based diagnostic definitions may introduce misclassification bias (eg, inclusion of non-VM cases), which can increase between-study heterogeneity and affect the generalizability of pooled estimates. Several choices strengthen the credibility of the findings. First, we harmonized results across different cognitive instruments by using Hedges’ g and applying a prespecified hierarchy that prioritized global screening measures, which reduced the risk of double-counting and limited construct drift. Second, the primary association was robust in sensitivity analyses: when we excluded studies requiring median-to-mean/SD conversion, the pooled deficit remained close to −1.0 with essentially no heterogeneity, and leave-one-out analyses preserved both the direction and the overall magnitude of effect. Third, ROBINS-I appraisal indicated predominantly Low-to-Moderate risk of bias in the domains most relevant to observational inference, and the single study judged to have Serious risk due to reliance on self-reported cognition did not contribute to the continuous meta-analyses.

Clinically, the present data support routine cognitive screening in VM and a multidisciplinary approach to management. Brief executive-function measures (e.g., Stroop or Trail-Making) may complement global screening tools by detecting deficits that are likely to matter most for everyday functioning and safety (36). For patients with objective impairment, practical steps include counseling regarding driving and other safety-critical activities, optimizing migraine prophylaxis with careful attention to cognitive side-effect profiles, and referral for vestibular rehabilitation and, where appropriate, cognitive rehabilitation (37). As standardized cognitive measures are adopted more consistently in VM clinics, the precision of future meta-analyses should improve, enabling more reliable subgroup analyses by age, disease duration, vestibular laboratory phenotype, and comorbidity burden.

Overall, despite these methodological constraints of the current literature, the overall evidence indicates that VM is associated with clinically relevant reductions in global cognitive performance and marked impairment in executive control. Recognizing and measuring these deficits in routine care can help identify higher-risk patients, tailor counseling and rehabilitation, and support future mechanistic and interventional studies that evaluate not only symptom improvement but also cognitive outcomes.

### Limitations

Several limitations should be acknowledged. The evidence base remains small, observational, and largely cross-sectional, which restricts causal inference and does not allow robust time-course conclusions, including whether cognitive performance differs meaningfully between ictal and interictal states. Residual confounding is also probable: anxiety, depression, sleep disturbance, medication effects (e.g., anticholinergic burden, migraine prophylaxis), and vestibular disability severity may each influence cognitive outcomes independent of VM (38), and as mapped out in our prevalence analysis, the primary studies largely failed to statistically adjust for these confounders when reporting overall impairment rates. Measurement heterogeneity is another key consideration. Although pooling with SMD facilitates integration across different scales to assess a common cognitive construct, pooling non-identical Stroop implementations (e.g., interference indices vs. total time) remains statistically precarious. This methodological compromise directly drove the substantial heterogeneity (I^2^ = 79%) observed in our executive function analysis, reaffirming that the pooled Stroop SMD should be viewed as an exploratory finding (39). For prevalence estimates, cross-study comparability is further limited by differing ACE-R impairment thresholds and by cultural and education-related effects on screening performance. Finally, the PubMed-only search strategy, while appropriate for a rapid synthesis of a narrowly framed question, may have missed studies indexed exclusively in Embase or Web of Science, and publication bias could not be meaningfully assessed given the small number of included studies.

These limitations highlight several priorities for future research. Prospective, adequately powered studies are needed that (1) use standardized cognitive test batteries with clear reporting of means, standard deviations, and impairment thresholds, (2) clearly distinguish between ictal and interictal testing to account for state-related effects, (3) include disease control groups, such as patients with migraine without vestibular symptoms, to separate migraine-related from vestibular-specific cognitive effects, and (4) systematically measure and adjust for key confounders, including mood symptoms, sleep disturbance, medication burden, and objective vestibular test findings (40–42). In addition, multimodal studies that combine behavioral cognitive assessment with vestibular function measures (eg, canal paresis and ocular motor indices) and network-level imaging may help clarify underlying mechanisms and identify clinically relevant treatment targets. Finally, interventional studies, including pharmacologic treatment, vestibular rehabilitation, and combined cognitive-vestibular training, should prespecify cognitive outcomes and include sufficient follow-up to determine the durability of any observed benefits.

## Conclusion

Current evidence from this focused synthesis indicates that adults with VM may experience clinically relevant cognitive impairment, including executive dysfunction, relative to healthy controls, with preliminary estimates suggesting up to 74% meet study-defined impairment thresholds. Therefore, clinicians may consider incorporating cognitive evaluation into routine VM assessment and management. Future research should prioritize harmonized cognitive outcomes and multimodal mechanistic studies. Potential contributing pathways include altered vestibulo-thalamo-cortical/frontoparietal network function and neuroinflammatory or immune mechanisms shared with migraine; accordingly, longitudinal studies should jointly measure cognition, vestibular phenotype, and inflammatory biomarkers to clarify mediation and to evaluate treatment responsiveness.

## Data Availability

The original contributions presented in the study are included in the article/Supplementary material, further inquiries can be directed to the corresponding author.

## Glossary

ACE-R: Addenbrooke’s Cognitive Examination-Revised
BAEP: brainstem auditory evoked potentials
BEST: Balance Evaluation Systems Test
CI: confidence interval
EEG: electroencephalography
HC: healthy control(s)
ICHD: International Classification of Headache Disorders
ICVD: International Classification of Vestibular Disorders
INPLASY: International Platform of Registered Systematic Review and Meta-analysis Protocols
JLO: Judgment of Line Orientation
MMSE: Mini-Mental State Examination
MoCA: Montreal Cognitive Assessment
MRI: magnetic resonance imaging
PRISMA: Preferred Reporting Items for Systematic Reviews and Meta-Analyses
RBANS: Repeatable Battery for the Assessment of Neuropsychological Status
REML: restricted maximum likelihood
ROBINS-I: Risk Of Bias In Non-randomized Studies - of Interventions
rs-fMRI: resting-state functional magnetic resonance imaging
SD: standard deviation
SMD: standardized mean difference
SPECT: single-photon emission computed tomography
TMT: Trail Making Test
VM: vestibular migraine
